# Isolation and antioxidant characterization of theaflavin for neuroprotective effect in mice model

**DOI:** 10.1002/fsn3.3337

**Published:** 2023-03-28

**Authors:** Arslan Ahmad, Farhana Nosheen, Muhammad Umair Arshad, Farhan Saeed, Muhammad Afzaal, Fakhar Islam, Ali Imran, Rabia Noreen, Yuosra Amer Ali, Mohd Asif Shah

**Affiliations:** ^1^ Department of Home Economics Government College University Faisalabad Faisalabad Pakistan; ^2^ Department of Food Science Government College University Faisalabad Faisalabad Pakistan; ^3^ Department of Food Sciences, College of Agriculture and Forestry University of Mosul Mosul Iraq; ^4^ University School of Business, Chandigarh University Mohali Punjab India

**Keywords:** inflammatory response, neuroprotective effect, oxidative stress, theaflavin

## Abstract

The mandate of the current investigation was to elucidate the therapeutic and antioxidant perspective of black tea. Purposely, black tea compositional analysis followed by polyphenol extraction and antioxidant characterization was done. Moreover, the theaflavin from black tea extract was also isolated through the solvent partition method. Lastly, the neuroprotective effect of isolated theaflavin was assessed through a bio‐efficacy trial. The outcomes delineated that black tea showed promising nutritional composition with special reference to protein and fiber. Among the extraction solvent, ethanol performed better as compared to methanol and water likewise, higher extraction was noticed at 60 min followed by 90 and 30 min. All the extracts indicated antioxidant activity reflected through significant DPPH, TPC, FRAP, and beta carotene as‐69.13 ± 3.00, 1148.92 ± 14.01, 752.44 ± 10.30, and 65.74 ± 3.28, respectively. However, isolated theaflavin exhibited higher antioxidant capacity as‐737.74 ± 12.55, 82.60 ± 2.33, and 853.77 ± 9.55, for TPC, DPPH, and FRAP, respectively, as compared to extracts. In 15 days' efficacy was physically induced with sciatic nerve injury h sciatic nerve injury physically and treated with isolated theaflavin. A total of 12 healthy albino mice were randomly assigned to either the control (*n* = 6) or theaflavin (5.0 mg/kg (*n* = 6)) groups. In these groups, behavioral tests were used to assess and compare enhanced functional recovery as well as skeletal muscle mass measurement. Serum samples included oxidative stress markers. In theaflavin leaves, behavioral tests revealed a statistically significant (p < .001) improvement in sensorimotor function restoration, muscle mass restoration, a substantial decrease in TOS, a significant increase in TAC, and enhanced antioxidative enzyme activity. Considering the above‐mentioned therapeutic perspectives of theaflavin, the current research was planned to optimize the isolation of theaflavin from black tea and probed for their neuroprotective effect in mice models.

## Graphical abstract

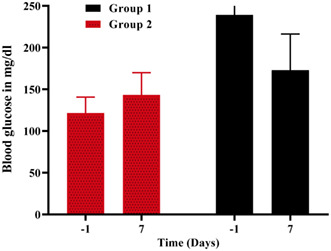

## 1 INTRODUCTION

Globally, oxidative stress caused numerous health disparities, directly and indirectly, owing to its adverse effect on human metabolism (Afzaal et al., 2023; Maqbool et al., 2023). Among the various curative strategies, polyphenol‐based dietary interventions are in limelight due to their capacity to normalize the imbalance between antioxidants and free radicals thus curtailing the menace of oxidative stress. Polyphenols are classified into four primary groups: flavonoids, lignans, stilbenes, and phenolic acids. Many in vivo and in vitro investigations have been conducted to assess their health consequences. They play a crucial function in defending the body against external stressors and removing reactive oxygen species (ROS), which are the root of many disorders (Islam et al., 2023). Polyphenols, which could be present in tea, cocoa, fruits, and vegetables, have the power to improve human health (Rana et al., 2022). Polyphenols have antioxidant, anti‐inflammatory, anti‐apoptotic, anticarcinogenic, and antibacterial properties and might be employed in medications, cosmetics, nutraceuticals, and food (Rajha et al., 2022). In humans, tea and coffee contain polyphenolic compounds that have antioxidant and neuroprotective properties (LIczbiński & Bukowska, 2022). They can also boost conception and help with Covid‐19 treatment. Because the brain has fewer antioxidant qualities than other organs, large levels of ROS appear to be more common, raising the risk of neurodegenerative diseases including Alzheimer's and Parkinson's (LIczbiński & Bukowska, 2022).

Tea is among the world's most nutritious beverages, and its aroma is an important component of the tea flavor profile as well as a significant predictor of tea quality (Li et al., 2013). The most common variety of tea is black tea, which accounts for 78% of global production (Chung, 2009; Imran et al., 2019). Tea consumption has been linked to oxidation, anti‐inflammation, and cancer prevention (de Majia et al., 2009), cardiovascular preventative medicine, and other health advantages (Stangl et al., 2007). Coffee has traditionally been linked to improved information, including brain clarity, focus, and relaxation. Several studies have discovered that the nutritional components of tea, notably caffeine, and the amino acid L‐theanine, change brain activity and have a positive impact on mental states such as emotional wellness and cognitive performance (Bryan, 2008). Parkinson's disease and Alzheimer's disease became two of the major neurodegenerative conditions worldwide (Mazumder & Choudhury, 2019; Zhou et al., 2019). Parkinson's disease affects 1–2% of the aging population, and the number of cases increases each year (Tysnes & Storstein, 2017). Alzheimer's disease impacted 35–40% of individuals over the age of 80, and its prevalence was rapidly increasing as the country's population aged (Chen et al., 2018; Mazumder & Choudhury, 2019). Tea may reduce the mortality rate of Parkinson's and Alzheimer's disease, as well as the risk of suicide, by reducing free radical precursors and inflammatory processes, changing variations, regulating the HPA axis and serotonin levels, and using metal‐chelating chemicals (Chen et al., 2018; Dong et al., 2015; Zhu et al., 2012). According to psychological studies, theaflavins protect the brain from long‐term damage (Anandhan et al., 2013). Theaflavins improved the behavior problem in an open area, rotary, and suspension tests (Anandhan et al., 2012). One study of 278 Parkinson's disease patients discovered that drinking more than three cups of tea each day could postpone the disease by 7.7 years (Kandinov et al., 2009). Pesticides and metal ions can cause peroxidation in the brain, which can cause dopamine cell death in Parkinson's disease patients (Chin‐Chan et al., 2015; Maturana et al., 2015). In the treatment of Parkinson's disease, tea has been proven to have neuroprotective qualities (Aquilano et al., 2008; Deb et al., 2019). Microglial cell activation was assumed to be important in selective neuron destruction in degenerative illnesses like Parkinson's disease (Li et al., 2004). Theaflavin from black tea (10 mg/kg) reduced the MPTP‐induced release of proinflammatory cytokines such as IL‐1β, IL‐6, TNF‐α, and IL‐6 in the striatum, showing that theaflavin's survival benefit is due to part to lower MPTP‐related neuron activation (Anandhan et al., 2013; Lagha & Grenier, 2016). Black tea extract upped the number of remaining tyrosine carbonic anhydrase immunoreactive neurons, the amount of tyrosine carbonic anhydrase protein, and the synthesis of tyrosine hydroxylase mRNA in the substantia nigra, all of which resulted in neuronal cell preservation (Chaturvedi et al., 2006). Theaflavin can diminish probenecid‐induced mortality by reducing apoptotic markers such as caspase‐3,8,9 (Anandhan et al., 2012). In an analysis of 26 epidemiological studies, tea drinking was significantly associated with a decreased frequency of impairments (Ma et al., 2016). Nevertheless, a study of 9375 Chinese people found that drinking black tea improved mental function but not green tea (Shen et al., 2015). Tea has been demonstrated to be a good source of ace inhibitors, which may help in Alzheimer's disease therapy (Baranowska‐Wojcik et al., 2020). Moreover, in Alzheimer's disease rats induced by aluminum chloride, black tea extract significantly improved cognitive deficits while decreasing the activity of (AChE) acetylcholinesterase (Mathiyazahan et al., 2015). Considering the above‐mentioned therapeutic perspectives of theaflavin, the current research was planned to optimize the isolation of theaflavin from black tea and probe for its neuroprotective effect in mice models.

## 2 MATERIALS AND METHODOLOGY

The present research was carried out in the nutritional laboratory of the department of food science, whereas an efficacy trial was conducted in the department of physiology. Black tea is produced in Faisalabad. The reagents and standards were supplied by Merck and Sigma‐Aldrich. Mice will be housed in the GCUF Physiology Department's Animal Room for the efficacy test. After inducing the sciatic nerve mechanical crush, two rat groups were created. The control group received regular rodent chow, whereas the other group received theaflavin extract mixed chow. The utility of behavioral analysis will be assessed.

The therapeutic potential of the examined medications against sciatic nerve injury was assessed using an efficacy trial. For this purpose, 12 mice were housed in the Animal House of the Department of physiology at GCUF, Faisalabad. The GCUF Departmental Ethical Committee accepted the procedure for this biological experiment, which satisfied ERC NO 2121 worldwide standards. For 2 weeks, the mice were fed a basic meal to help them adjust to their new surroundings. During the experiment, the temperature (22°C) and relative humidity (55%) will be monitored, as well as a 12‐h light–dark cycle.

## 3 THE CHARACTERISTICS OF BLACK TEA

At first, black tea was tested for configurational characteristics such as causal test, nutrient biomarker, nutrient characteristics, toxic metabolites, and total phenolic excavation.

## 4 EVALUATION OF CONSTITUENTS

Black tea tests were performed in triplicate for compositional analysis on a dry‐weight basis.

## 5 POLYPHENOL EXTRACTION

Tea polyphenols were derived in three different time intervals of 30, 60, and 90 min at a safe temperature of 60°C that used water, methanol, and ethanol process. After that, the sample was obtained through a muslin cloth, and the fluids were retrieved by rotary and freeze‐drying. Table 1 shows the method for extraction.

**TABLE 1:** Procedure used to calculate extraction yield.

Treatment	Solvent	Time (minutes)
T1	Water (100%)	30
T2	Methanol (%)	60
T3	Ethanol (%)	90
T4	Water (%)	30
T5	Methanol (%)	60
T6	Ethanol (%)	90
T7	Water (100%)	30
T8	Methanol (100%)	60
T9	Ethanol (100%)	90

## 6 ISOLATION OF THEAFLAVIN

It will be carried out at a 1:6 ratio at 30‐, 60‐, and 90‐min intervals, water will be used to extract theaflavin, and theaflavin will be separated using the solvent partition technique (Xie et al., 1993). With exception of liquid extracts, all extracts will be concentrated in boiling water, sifted, and dissolvable partitioned with chloroform, ethyl acetate, and butanol. Once the theaflavin‐rich fractions are separated, freeze‐drying (CHIRST, Alpha 1–4 LD plus, Germany) would be used.

## 7 BIOLOGICAL TEST

Theaflavin's therapeutic efficacy in the treatment of sciatic nerve injury was to be evaluated through an efficacy study. Male albino mice weighing 25–35 g were purchased from the Department of Physiology at Government College University Faisalabad's animal treatment facility. The creatures were kept in cages, one per cage (plastic rodent cage). Housing requirements such as room temperature of 23–27°C, supply of a 12‐h light and 12‐h dark cycle, sufficient moisture, and ad libitum delivery of diet and drinking water were maintained during the acclimatization period (1 week) and the whole experiment. All behavioral observations and experiments were conducted throughout the day.

## 8 BEHAVIORAL ANALYSES

### 8.1 Nociceptive analysis

The pinprick test is another method for assessing sensory capacity recovery after nerve damage. It was carried out by the procedure outlined by (Chen et al., 2018).

### 8.2 Walking track analysis

The sciatic functional index (SFI) is a numerical method for evaluating the motor functions of mice. The restoration of motor functions following sciatic nerve crush was investigated by calculating the SFI based on walking track analysis, as stated in previous studies by Komirishetty et al. (2016).

### 8.3 Grip strength analysis

Resistance training testing is an efficient method of assessing motor function recovery after a sciatic crush injury. The trying to comprehend the strength of both hind limbs (upper distal phalanx and caudal to the affected site) was evaluated for each mouse using a grip trimeter (Bioseb, Chaville, France). Its final result was calculated using the average of three readings taken at 1–2 min intervals (Hussain et al., 2013; Razzaq et al., 2020).

### 8.4 Muscle weight analysis

Muscular atrophy occurs when the connection between the muscle and the nerve is disrupted as a result of an injury, resulting in muscle mass. As a result, muscle mass evaluation provides a method for determining the degree of muscular atrophy, which is a major barrier to functional status. The soleus and tibialis anterior (TA) muscles were removed and weighed from both rear legs (upper extremity and contralateral to the affected site). In the same animals, the muscular proportion was computed by dividing the mass of the contralateral muscular even by the weight of the ipsilateral lean muscle. To assess operational retrieval, the mean ratio was calculated for each group and compared across groups (Hagstrom et al., 2004).

### 8.5 Random glycemic level

The random glycemic data were measured throughout nerve impingement initiation to investigate the role of glucose in enraging neurotic episodes in the nervous injured area. Hyperglycemia inhibits its healing process after injuries. In both groups, the glucose level was evaluated by depositing a sample of spider tail blood on a blood sugar meter strip and measuring it with an Accu‐Check glucometer, as previously stated (Asmat et al., 2016; Razzaq et al., 2020).

## 9 BIOCHEMICAL ANALYSES

### 9.1 Total antioxidant capacity

A body system's antioxidant activities are defined as its ability to confront free radicals produced as a result of various pathogenetic processes that occur in the body. An ideal antioxidant‐bearing capacity improves the body's ability to resist various ailments (Erel, 2004).

### 9.2 Total oxidant status

The degree of Total oxidant status (TOS) within a living system is reported to be related to the stage of oxidative stress. This test determines the overall state of oxidative stress. This was achieved by utilizing the protocol outlined in previous studies (Aziz et al., 2019; Erel, 2005).

## 10 STATISTICAL ANALYSIS

SAS was utilized to evaluate the data gathered throughout this investigation (version 9.1; Cary, NC). A two‐way ANOVA was used to assess the effect of extreme action period and dissolvable on polyphenol isolation (ANOVA). Furthermore, in the efficacy study, ANOVA and LSD were used to assess the effectiveness of therapies using Graph Pad prism 8.4.2.

## 11 RESULTS AND DISCUSSION

Proximate analysis is a critical factor in determining raw material quality. Black tea (dry weight basis) was subjected to different quality traits assessment and revealed moisture, crude fat, crude protein, crude fiber, ash, NFE, and total alkaloids as 7.02 ± 0.21, 4.60 ± 0.20, 15.13 ± 0.70, 15.30 ± 0.76, 4.87 ± 0.22, 53.15 ± 2.01, and 2.51 ± 0.08, respectively (Table 2).

**TABLE 2:** Characteristics of black tea analysis.

Proximate analysis of black tea	
Parameters	Quantity
Moisture (%)	7.02 ± 0.21
Ash (%)	4.87 ± 0.22
Crude fat (%)	4.60 ± 0.20
Crude fiber (%)	15.30 ± 0.76
Crude protein (%)	15.13 ± 0.70
NFE	53.15 ± 2.01
Total alkaloids	2.51 ± 0.08

*Note*: Values are expressed as means ± standard deviation (*n* = 3).

**FIGURE 1:**
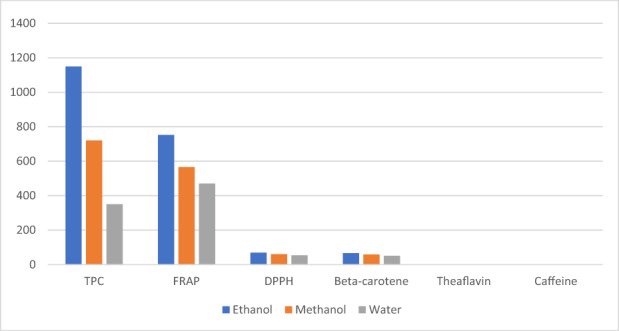
Effect of solvent on black tea extract.

**FIGURE 2:**
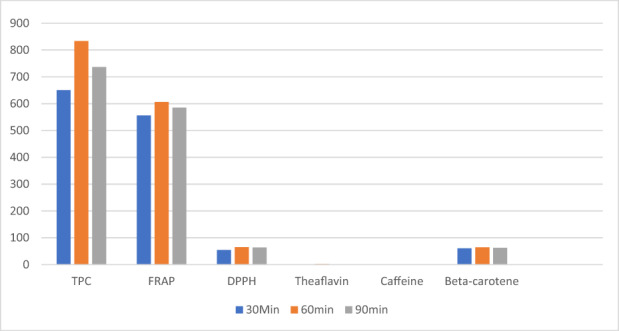
Potential of time on black tea extract.

**FIGURE 3:**
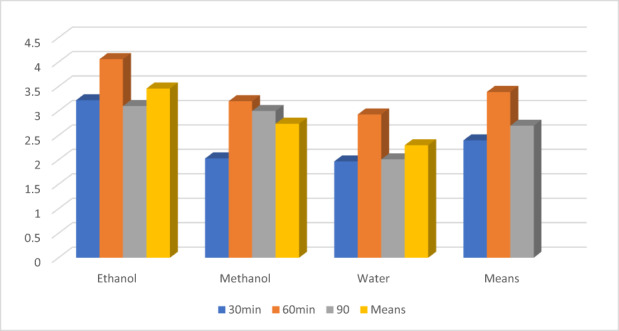
Extraction yield of theaflavin.

The findings of the current investigation regarding the proximate profile are in line with the earlier findings of Aregbesola et al. (2018) determined moisture, ash, protein, and fiber levels in black tea samples as 5.94, 5.46, 11.03, and 14.25, respectively. Earlier, Modupe et al. (2013) determined moisture, ash, crude fat, crude protein, and nitrogen levels in black tea were 8.10, 9.59, 3.25, 17.78, and 2.83%, respectively. According to Ramdani et al. (2018), green tea alkaloids (31.5%) are significantly higher than black tea alkaloids (28.7%). The alkaloids result was statistically significant when compared with the previous findings of Erol et al. (2010), who discovered total alkaloids ranging from 25.97 to 26.26 mg/g in various Turkish black tea samples. The antioxidant properties of black tea extracts are affected by the solvent used and the time of extraction. Increasing the extraction time from 30 to 60 min increased antioxidant activity in all extracts; however, at 90 min, antioxidant activity decreased. The ethanolic extract significantly outperforms the water and methanolic extracts shown in Figures 1, 2, 3.

The means for solvents regarding antioxidants indices (Figure 3) showed the highest values for an ethanolic extract for TPC (1148.92 ± 14.01a), FRAP (752.44 ± 10.30), DPPH (69.13 ± 3.00) ß‐carotene (65.74 ± 3.28), theaflavin (2.52 ± 0.12), and caffeine (1.88 ± 0.06), respectively, as compared to the methanol and water. The methanolic extract performs better than water as 720.41 ± 10.23, 565.78 ± 11.01, 60.37 ± 2.00, 58.87 ± 2.94, 2.04 ± 0.10, and 1.70 ± 0.07, respectively, for these traits. Likewise, the lowest values were recorded in water extract 350.02 ± 5.12, 469.78 ± 10.50, 53.27 ± 3.10, 50.27 ± 3.80, 1.22 ± 0.01, and 1.55 ± 0.08, respectively.

**FIGURE 4:**
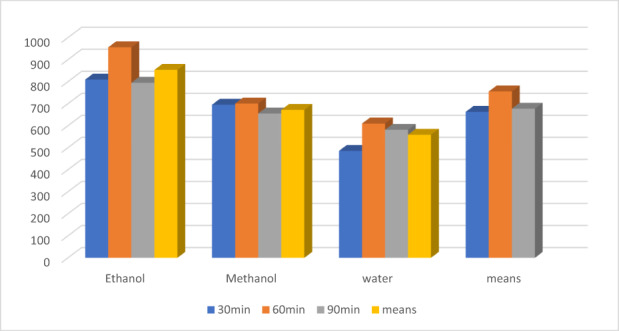
FRAP (theaflavin) μmol Fe2+/g.

Similarly, time (Figure 4) also showed good performance at 60 min for TPC (833.30 ± 20.1a mg/100 g GAE), FRAP (605.89 ± 15.904a μmol Fe2+/g), DPPH (65.30 ± 3.00a %), ß‐carotene (64.24 ± 3.60a %), theaflavin (2.00 ± 0.13a %), except for caffeine (1.67 ± 0.04a %). However, the extracts at 90 min showed maximum caffeine (1.74 ± 0.05b %). Likewise, the extracts at 30 min showed minimum values for TPC, FRAP, DPPH, ß‐carotene, theaflavin, catechins, and caffeine by 650.44 ± 15.01c mg/100 g GAE, 556.00 ± 13.01c μmol Fe2+/g, 59.69 ± 2.30%, 60.30 ± 2.80c, 1.69 ± 0.04c, and 1.63 ± 0.01, respectively.

**FIGURE 5:**
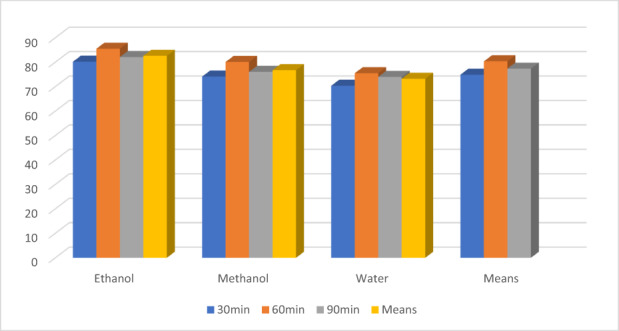
DPPH activity (theaflavin)%.

Extraction efficiency was influenced by time and the maximum yield for theaflavin was obtained at 60 min 3.39 ± 0.17 g/100 g, while, minimum at 30 min 2.40 ± 0.12, respectively. However, at 90 min, the values were 2.71 ± 0.14 for theaflavin (Figure 5).

Yang et al. (2008) showed that 1.51 g/50 g of theaflavin in black tea samples which is comparable with theaflavin yield results. They used ethyl alcohol as an extraction fluid during the experiment. The difference in extraction yields in this test is due to the incorrect assumption that extraction times were 40 min rather than 60 min. Tea polyphenols recovered 75–80% of their isolation, according to Sharma and Zhou (2011).

The theaflavin showed high value for FRAP in ethanol than for methanol and water. The FRAP values of theaflavin in ethanolic, methanolic, and water extract were 853.77 ± 9.55, 672.11 ± 12.33, and 559.19 ± 11.22 μmol Fe2+/g, shown in Figure 4, respectively. Similarly, for time intervals, theaflavin showed higher FRAP activity at 30 min (663 ± 10.33 μmol Fe2+/g), 60 min (755.86 ± 8.33 μmol Fe2+/g), and 90 min (677.55 ± 9.34 μmol Fe2+/g). Likewise, the statistical analysis showed the momentous effect of time and solvents for DPPH. Figure 5 showed the highest values for DPPH in theaflavin in ethanolic extract 82.60 ± 2.33%, as compared to the methanol 76.73 ± 3.23%, and water 73.23 ± 2.63%, correspondingly. Time also showed the highest DPPH activity of theaflavin was 80.36 ± 4.00% at 60 min, while 77.36 ± 3.35 and 74.89 ± 3.62% at 90 min and 30 min, respectively.

**FIGURE 6:**
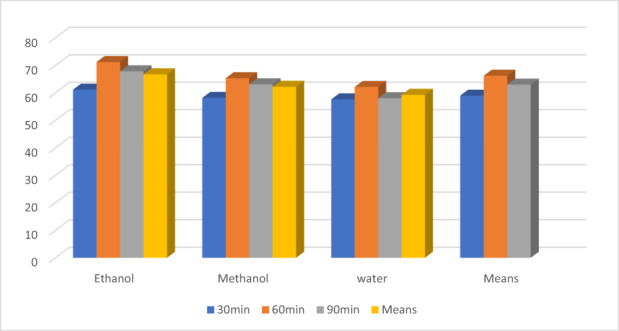
ß‐carotene (theaflavin)%.

Furthermore, statistical analysis elucidated the significant effect of solvent and time for the ß‐carotene activity of theaflavin presented in Figure 6. Similarly, ethanolic extract showed maximum activity of 66.74 ± 3.22% than methanol 62.20 ± 2.88% and water 59.27 ± 2.55%. Time also showed that 60‐min extraction time resulted in higher ß‐carotene activity 66.24 ± 2.15% in comparison with 90 and 30 min by 63.00 ± 3.00 and 58.97 ± 2.44%, respectively.

**FIGURE 7:**
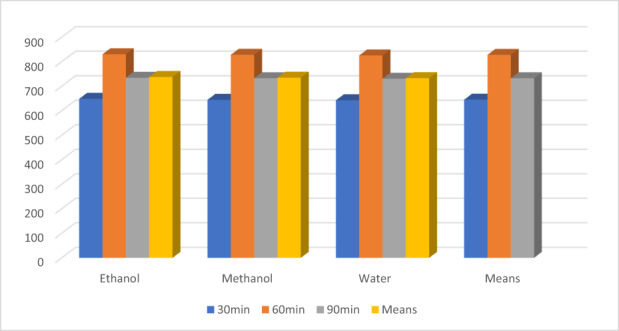
TPC mg/100 g GAE.

The statistical analysis shown imparted a significant effect of solvents and time on the yield of TPC. Theaflavin showed TPC values maximum for ethanol as compared to methanol and water. Figure 7 showed higher activity for theaflavin. The TPC values in ethanolic were 737.74 ± 12.55, followed by methanol and ethanol 735.08 ± 10.22 and 732.77 ± 9.99 mg/100 g GAE, correspondingly. However, for time intervals, theaflavin showed higher TPC activity at 60 min (827.99 ± 15.55 mg/100 g GAE). While at 30 min, theaflavin showed minimum value (645.10 ± 12.44 mg/100 g GAE), and at 90 min, theaflavin showed732.55 ± 8.55 mg/100 g GAE.

**FIGURE 8:**
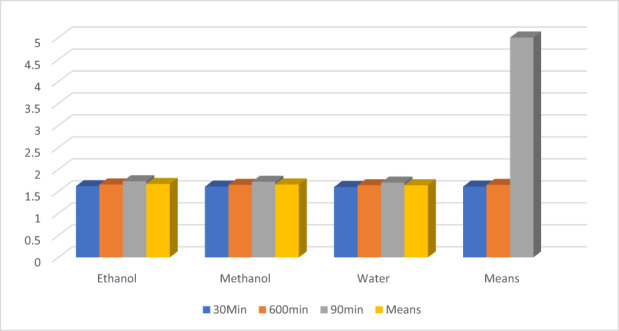
Caffeine %.

The statistical analysis shown elucidated a significant effect of solvents and time on the yield of Caffeine. The value for Caffeine% activity of theaflavin showed a maximum in ethanol, while the minimum for water is shown in Figure 8. In theaflavin, ethanolic extract showed maximum activity of 1.67 ± 0.07% than methanol 1.66 ± 0.06% and water 1.64 ± 0.05%. Furthermore, 90‐min extraction time resulted in higher Caffeine activity by 1.71 ± 0.04% in comparison with 60 and 30 min by 1.65 ± 0.02 and 1.61 ± 0.01%, respectively. Initially, Sun et al. (2012) investigated the DPPH free radical scavenging capacity of an Assam black tea extract (ASTE) and theaflavin combination in vitro, and their results are in resemble to the most recent DPPH free radical scavenging activity findings (TFSM). They discovered that TFSM has a higher DPPH value than ASTE (60–97%) (40–82%). Initially, Friedman et al. (2006) created bioactive fragments with aqueous ethanol and water at different solvent‐to‐water ratios of 60%, 70%, and 80%. TF1, TF2, TF2B, and TF3 levels in ethanolic extract ranged from 1.7 to 4.4, 3.6 to 5.8, 0.4 to 1.8, and 1.6 to 5.12 mg/g, respectively. The affiliated subsets in water extract ranged from 0.1 to 1.1, 1.2 to 2.1, 0.2 to 1.10, and 1.1 to 3.21 mg/g. According to the most recent research, ethanol and methanol produce higher polyphenolic yields of tea catechins than water, which support the findings on the effect of various solvents on tea polyphenol extraction (Bastos et al., 2007). The study's findings are aligned with those of another research study conducted by (Chen et al., 2012).

## 12 BIOLOGICAL TRAILS

**FIGURE 9:**
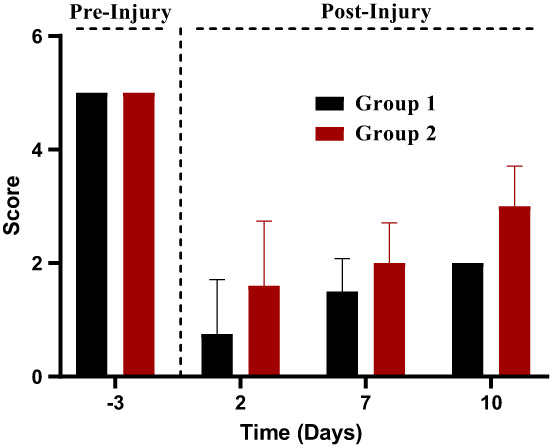
Paw withdrawal latency.

Statistical analysis elucidated the significant effect of treatment and study interval on the pinprick test of nerve injury in mice. Similarly, Figure 9 shows that the sensory threshold was assessed using pinprick analysis, and the treatment group produced significantly higher scores in response to the pinprick stimulus. On day 7, the 5.0 mg/kg group appeared to be significantly (*p* = .01) effective, and on day 10, it appeared to be highly effective (*p* = .0001). As a result, statistically significant improvements in outcomes, as shown in Figure 9, were observed. These findings support theaflavin's ability to lower sensory threshold following sciatic nerve injury.

**FIGURE 10:**
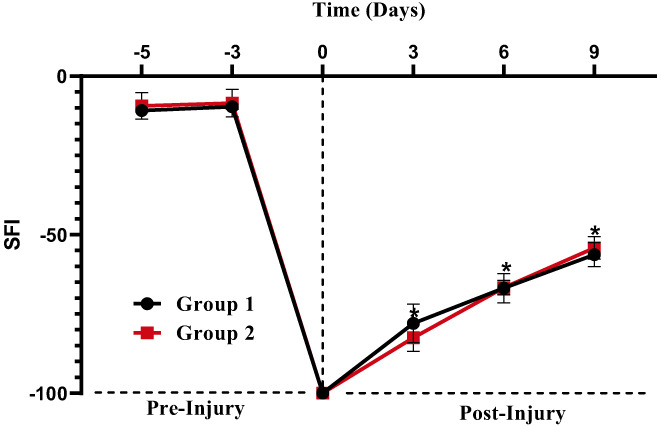
Sciatic functional index.

Despite technological advancement, humanity has been plagued by a wide range of diseases. Regulating and tracking cases of life‐long disability or biological reliance caused by nerve damage caused by various types of crashes, most notably roadside motor vehicle crashes, gunfire, and sudden falls, is one of the most difficult issues in the field of health research (Kouyoumdjian et al., 2017). Unfortunately, these incidents are one of the leading causes of PNI development, especially in over‐packed, stunted countries like Pakistan, where the transportation system is already overburdened (Mushtaq et al., 2021). The pinprick test was used to measure sensory function recovery after sciatic nerve injury. This memory test tests mice's sensory threshold retrieval and our results are in line with his findings (Deuis et al., 2017). One of these critical features is oxidative stress, which greatly contributes to the pathophysiological processes that occur at the injury site (Wang et al., 2015). The reaction spirals which include mitochondrial disorders, dementia, neuronal damage, and apoptosis (Hussain et al., 2020) induce systemic nerve damage, produce oxidants that aggravate the harm, and delay diagnosis (Al‐Nimer et al., 2012; Areti et al., 2014). When compared with the control group, the treatment group demonstrated substantial improvement in motor functions in terms of SFI measurement and grip strength force (percent of original force) (Figure 10). According to SFI results, animals in the treatment group showed a considerably better walking pattern. In this study, the 5.0 mg/kg groups were notably efficient at normalizing SFI levels on day 6 (*p* = .001) and day 9 (*p* = .006) after injury. These data suggest that theaflavin can hasten motor functional recovery.

**FIGURE 11:**
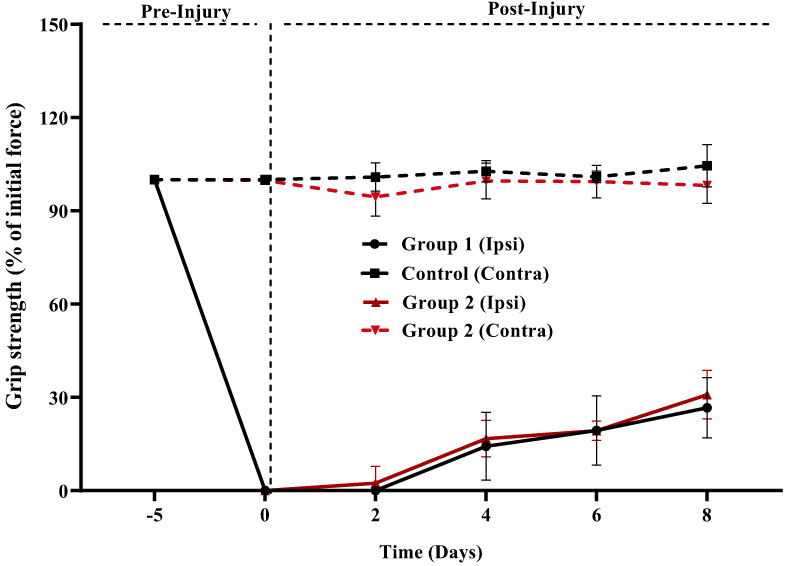
GRIP strength.

**FIGURE 12:**
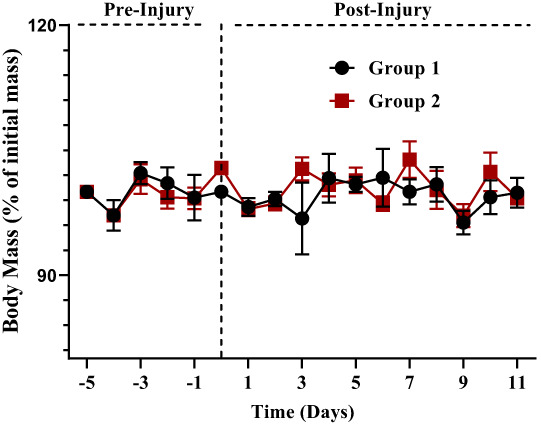
Body mass.

When compared with the control group, all treatment groups improved significantly in terms of grip strength force (percent of beginning force) shown above in (Figure 11). When compared with controls on day 6 post‐injury, treatment groups showed a significant difference (rapid restoration of the gripping ability of injured paw to hold the wire of grip strength meter), with the 5.00 mg/kg group revealing to be suggestively advantageous (*p* = .001). In the following periods, the treatment group had significantly greater gripping strength disparities (*p* = .001) than the control group (days 7 and 8 post‐injury). The 5 mg/kg group, on the other hand, demonstrated significant changes on day 8 following injury (*p* = .02). Our results are in line with the findings of (Rasul et al., 2019), for motor functions and retrieval in case of peripheral nerve injury. Their results indicate that the treated group acquires motor functions earlier as compared to Normal control group. During the whole experiment, the comparison of body mass % among all groups was similarly determined to be non‐significant (*p* = .956) (Figure 12).

**FIGURE 13:**
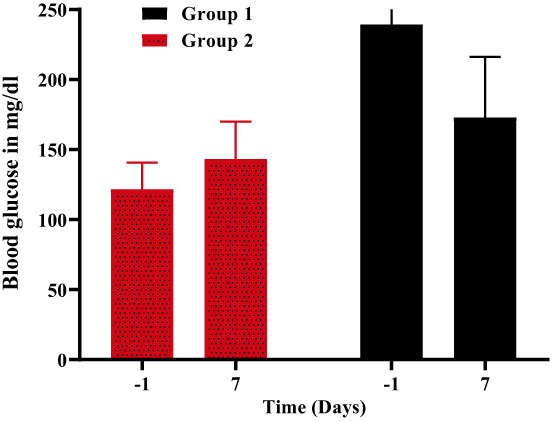
Glycemic level.

The statistically significant drop in glycemia following theaflavin‐containing diet consumption illustrates the hypoglycemic influence of this regimen. In this context, the glycemic levels of all groups were evaluated and compared before and after injury induction (Figure 13). It was revealed that all treatment groups kept their glucose levels regular. However, mice fed regular chow had somewhat higher glucose levels (control group). At 5.00 mg/kg, theaflavin significantly lowered glycemic levels (*p* = .002).

**FIGURE 14:**
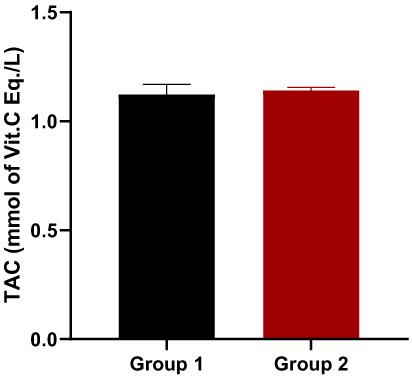
Total antioxidant capacity level.

**FIGURE 15:**
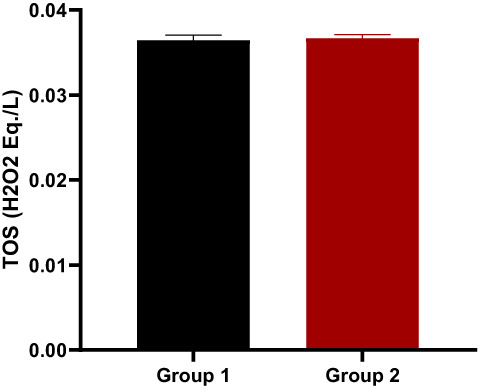
Total oxidant status level.

Total antioxidant capacity (TAC) and TOS levels in animal blood samples were measured following decapitation to best explain the mechanisms involved in enhanced functional recovery, and the results demonstrated that theaflavin leaves had a highly significant influence on reducing oxidative stress (i.e., improved TAC values and lowered TOS values). TAC levels increased significantly (p.0001) in all treated groups as compared to the control (Figure 14), confirming theaflavin's outstanding antioxidant activity. The number of free radicals increases when the sciatic nerve is injured, raising the TOS level. TOS values in all treated groups were significantly lower (p.0001) than in the control group (Figure 15). However, these results are in line with the findings of (Rasul et al., 2019).

## 13 CONCLUSION AND RECOMMENDATIONS

It is envisaged from the current findings that black tea effectively alleviates oxidative stress symptoms. Black tea is selected due to its high phenolic content and high antioxidant activity based on the DPPH, FRAP, and TPC. The results showed that black tea extracts performed better with ethanol solvent than with water or methanol. Similarly, antioxidant indices of isolated theaflavin imparted significant activity with ethanol at 60 min. In light of all of the above, the study concluded that theaflavin not only expedited functional sciatic nerve recovery, as seen by behavioral parameter data but also effectively combats oxidative stress. However, there is a dire need to investigate theaflavin for these health‐promoting effects on a molecular and genetic level. No doubt, this research provides a basic footstep toward the neuroprotective effect of theaflavin however, mechanistic elucidation on cellular and molecular levels is still lacking to unveil its application at a large level. It is recommended that human‐based clinical should be carried out in the future.

## FUNDING INFORMATION

The authors declare that no funds, grants, or other support were received during the preparation of this manuscript.

## CONFLICT OF INTEREST STATEMENT

The authors declare that they have no conflict of interest.

## ETHICS STATEMENT

This article does not contain any studies with human participants or animals performed by any of the authors.

## CONSENT TO PARTICIPATE

Corresponding and all the co‐authors are willing to participate in this manuscript.

## CONSENT FOR PUBLICATION

All authors are willing for publication of this manuscript.

## Data Availability

Even though adequate data have been given in the form of tables and figures, all authors declare that if more data are required, then the data will be provided on a request basis.

## References

[fsn33337-bib-0102] Afzaal, M. , Saeed, F. , Ateeq, H. , Akhtar, M. N. , Imran, A. , Ahmed, A. , Aamir, M. , Islam, F. , Yasmin, I. , Shah, Y. A. , Hussain, M. , Hameed, A. , Kumar, R. , & Awuchi, C. G. (2023). Probiotics encapsulated gastroprotective cross‐linked microgels: Enhanced viability under stressed conditions with dried apple carrier. Food Science & Nutrition, 11(2), 817–827. 36789050 10.1002/fsn3.3116 PMC9922151

[fsn33337-bib-0001] Al‐Nimer, M. , Al‐Ani, F. , & Ali, F. (2012). Role of nitrosative and oxidative stress in neuropathy in patients with type 2 diabetes mellitus. Journal of Neurosciences in Rural Practice, 3(1), 41–44. 10.4103/0976-3147.91932 22346190 PMC3271613

[fsn33337-bib-0002] Anandhan, A. , Essa, M. M. , & Manivasagam, T. (2013). Therapeutic attenuation of Neuroinflammation and apoptosis by black tea Theaflavin in chronic MPTP/probenecid model of Parkinson's disease. Neurotoxicity Research, 23(2), 166–173. 10.1007/s12640-012-9332-9 22669749

[fsn33337-bib-0003] Anandhan, A. , Tamilselvam, K. , Radhiga, T. , Rao, S. , Essa, M. M. , & Manivasagam, T. (2012). Theaflavin, a black tea polyphenol, protects nigral dopaminergic neurons against chronic MPTP/probenecid induced Parkinson's disease. Brain Research, 1433, 104–113. 22138428 10.1016/j.brainres.2011.11.021

[fsn33337-bib-0004] Aquilano, K. , Baldelli, S. , Rotilio, G. , & Ciriolo, M. R. (2008). Role of nitric oxide synthases in Parkinson's disease: A review on the antioxidant and anti‐inflammatory activity of polyphenols. Neurochemical Research, 33(12), 2416–2426. 10.1007/s11064-008-9697-6 18415676

[fsn33337-bib-0005] Aregbesola, O. A. , Faborode, M. O. , & Hounkanrin, B. A. (2018). Studies on black tea production from fresh roselle calyxes. International Food Research Journal, 25(1), 310–313.

[fsn33337-bib-0006] Areti, A. , Yerra, V. G. , Naidu, V. , & Kumar, A. (2014). Oxidative stress and nerve damage: Role in chemotherapy induced peripheral neuropathy. Redox Biology, 2, 289–295. 10.1016/j.redox.2014.01.006 24494204 PMC3909836

[fsn33337-bib-0007] Asmat, U. , Abad, K. , & Ismail, K. (2016). Diabetes mellitus and oxidative stress—A concise review. Saudi Pharmaceutical Journal, 24, 547–553. 10.1016/j.jsps.2015.03.013 27752226 PMC5059829

[fsn33337-bib-0008] Aziz, N. , Rasul, A. , Malik, S. A. , Anwar, H. , Imran, A. , Razzaq, A. , & Hussain, G. (2019). Supplementation of *Cannabis sativa* L. leaf powder accelerates functional recovery and ameliorates haemoglobin level following an induced injury to sciatic nerve in mouse model. Pakistan Journal of Pharmaceutical Sciences, 32(2), 785–792. 31103973

[fsn33337-bib-0009] Baranowska‐Wojcik, E. , Szwajgier, D. , & Winiarska‐Mieczan, A. (2020). Regardless of the brewing conditions, various types of tea are a source of acetylcholinesterase inhibitors. Nutrients, 12(3), 709. 10.3390/nu12030709 32155927 PMC7146204

[fsn33337-bib-0010] Bastos, D. H. , Saldanha, L. A. , Catharino, R. R. , Sawaya, A. , Cunha, I. B. , Carvalho, P. O. , & Eberlin, M. N. (2007). Phenolic antioxidants identified by ESI‐MS from yerba mate (*Ilex paraguariensis*) and green tea (*Camelia sinensis*) extracts. Molecules, 12(3), 423–432. 17851401 10.3390/12030423 PMC6149459

[fsn33337-bib-0011] Bryan, J. (2008). Psychological effects of dietary components of tea: Caffeine and L‐theanine. Nutrition Reviews, 66, 82–90. 18254874 10.1111/j.1753-4887.2007.00011.x

[fsn33337-bib-0012] Chaturvedi, R. K. , Shukla, S. , Seth, K. , Chauhan, S. , Sinha, C. , Shukla, Y. , & Agrawal, A. K. (2006). Neuroprotective and Neurorescue effect of black tea extract in 6‐hydroxydopamine‐lesioned rat model of Parkinson's disease. Neurobiology of Disease, 22(2), 421–434. 10.1016/j.nbd.2005.12.008 16480889

[fsn33337-bib-0013] Chen, H. , Zhang, Y. , Lu, X. , & Qu, Z. (2012). Comparative studies on the physicochemical and antioxidant properties of different tea extracts. Journal of Food Science and Technology, 49(3), 356–361. 23729856 10.1007/s13197-011-0291-6 PMC3614050

[fsn33337-bib-0014] Chen, S. Q. , Wang, Z. S. , Ma, Y. X. , Zhang, W. , Lu, J. L. , Liang, Y. R. , & Zheng, X. Q. (2018). Neuroprotective effects and mechanisms of tea bioactive components in neurodegenerative diseases. Molecules, 23(3), 512. 10.3390/molecules23030512 29495349 PMC6017384

[fsn33337-bib-0015] Chin‐Chan, M. , Navarro‐Yepes, J. , & Quintanilla‐Vega, B. (2015). Environmental pollutants as risk factors for neurodegenerative disorders: Alzheimer and Parkinson diseases. Frontiers in Cellular Neuroscience, 9, 124. 10.3389/fncel.2015.00124 25914621 PMC4392704

[fsn33337-bib-0016] Chung, S. Y. (2009). Cancer prevention by tea: Animal studies, molecular mechanisms and human relevance. Nature Reviews. Cancer, 9(6), 429–439. 19472429 10.1038/nrc2641 PMC2829848

[fsn33337-bib-0017] de Mejia, E. G. , Ramirez‐Mares, M. V. , & Puangpraphant, S. (2009). Bioactive components of tea: Cancer, inflammation and behavior. Brain, Behavior, and Immunity, 23(6), 721–731. 19258034 10.1016/j.bbi.2009.02.013

[fsn33337-bib-0018] Deb, S. , Dutta, A. , Phukan, B. C. , Manivasagam, T. , Thenmozhi, A. J. , Bhattacharya, P. , Paul, R. , & Borah, A. (2019). Neuroprotective attributes of L‐theanine, a bioactive amino acid of tea, and its potential role in Parkinson's disease therapeutics. Neurochemistry International, 129, 104478. 10.1016/j.neuint.2019.104478 31145971

[fsn33337-bib-0020] Deuis, J. R. , Dvorakova, L. S. , & Vetter, I. (2017). Methods used to evaluate pain behaviors in rodents. Frontiers in Molecular Neuroscience, 10, 284. 10.3389/fnmol.2017.00284 28932184 PMC5592204

[fsn33337-bib-0021] Dong, X. X. , Yang, C. , Cao, S. H. , Gan, Y. , Sun, H. L. , Gong, Y. H. , Yang, H. J. , Yin, X. X. , & Lu, Z. X. (2015). Tea consumption and the risk of depression: A meta‐analysis of observational studies. The Australian and New Zealand Journal of Psychiatry, 49(4), 334–345. 10.1177/0004867414567759 25657295

[fsn33337-bib-0022] Erel, O. (2004). A novel automated direct measurement method for total antioxidant capacity using a new generation, more stable ABTS radical cation. Clinical Biochemistry, 37(4), 277–285. 10.1016/j.clinbiochem.2003.11.015 15003729

[fsn33337-bib-0023] Erel, O. (2005). A new automated colorimetric method for measuring total oxidant status. Clinical Biochemistry, 38(12), 1103–11111. 10.1016/j.clinbiochem.2005.08.008 16214125

[fsn33337-bib-0024] Erol, N. T. , Sarı, F. , & Velioglu, Y. S. (2010). Polyphenols, alkaloids and antioxidant activity of different grades Turkish black tea. Gida, 35(3), 161–168.

[fsn33337-bib-0025] Friedman, M. , Levin, C. E. , Choi, S. H. , Kozukue, E. , & Kozukue, N. (2006). HPLC analysis of catechins, theaflavins, and alkaloids in commercial teas and green tea dietary supplements: Comparison of water and 80% ethanol/water extracts. Journal of Food Science, 71(6), C328–C337.

[fsn33337-bib-0055] Hagstrom, J. E. , Hegge, J. , Zhang, G. , Noble, M. , Budker, V. , Lewis, D. L. , Herweijer, H. , & Wolff, J. A. (2004). A facile nonviral method for delivering genes and siRNAs to skeletal muscle of mammalian limbs. Molecular Therapy, 10(2), 386–398. 15294185 10.1016/j.ymthe.2004.05.004

[fsn33337-bib-0027] Hussain, G. , Schmitt, F. , Henriques, A. , Lequeu, T. , Rene, F. , Bindler, F. , Dirrig‐Grosch, S. , Oudart, H. , Palamiuc, L. , Metz‐Boutigue, M.‐H. , Dupuis, L. , Marchioni, E. , Gonzalez De Aguilar, J.‐L. , & Loeffler, J.‐ P. (2013). Systemic down‐regulation of delta‐9 desaturase promotes muscle oxidative metabolism and accelerates muscle function recovery following nerve injury. PLoS One, 8(6), e64525. 10.1371/journal.pone.0064525 23785402 PMC3681796

[fsn33337-bib-0028] Hussain, G. , Wang, J. , Rasul, A. , Anwar, H. , Qasim, M. , Zafar, S. , Aziz, N. , Razzaq, A. , Hussain, R. , de Aguilar, J.‐L. , & Sun, T. (2020). Current status of therapeutic approaches against peripheral nerve injuries: A detailed story from injury to recovery. International Journal of Biological Sciences, 16, 116–134. 10.7150/ijbs.35653 31892850 PMC6930373

[fsn33337-bib-0104] Imran, A. , Butt, M. S. , Xiao, H. , Imran, M. , Rauf, A. , Mubarak, M. S. , & Ramadan, M. F. (2019). Inhibitory effect of black tea (Camellia sinensis) theaflavins and thearubigins against HCT 116 colon cancer cells and HT 460 lung cancer cells. Journal of Food Biochemistry, 43(5), e12822. 31353529 10.1111/jfbc.12822

[fsn33337-bib-0103] Islam, F. , Amer Ali, Y. , Imran, A. , Afzaal, M. , Zahra, S. M. , Fatima, M. , Saeed, F. , Usman, I. , Shehzadi, U. , Mehta, S. , & Shah, M. A. (2023). Vegetable proteins as encapsulating agents: Recent updates and future perspectives. Food Science & Nutrition, 11(3), 1–13. 10.1002/fsn3.3234 PMC10084973 37051354

[fsn33337-bib-0029] Kandinov, B. , Giladi, N. , & Korczyn, A. D. (2009). Smoking and tea consumption delay onset of Parkinson's disease. Parkinsonism & Related Disorders, 15(1), 41–46. 10.1016/j.parkreldis.2008.02.011 18434232

[fsn33337-bib-0030] Komirishetty, P. , Areti, A. , Yerra, V. G. , Pk, R. , Sharma, S. S. , Gogoi, R. , Sistla, R. , & Kumar, A. (2016). PARP inhibition attenuates neuroinflammation and oxidative stress in chronic constriction injury‐induced peripheral neuropathy. Life Sciences, 150, 50–60. 10.1016/j.lfs.2016.02.085 26921631

[fsn33337-bib-0031] Kouyoumdjian, J. , Graç, C. , & Ferreira, V. M. (2017). Peripheral nerve injuries: A retrospective survey of 1124 cases. Neurology India, 65(3), 551–555. 10.4103/neuroindia.NI_987_16 28488619

[fsn33337-bib-0032] Lagha, A. B. , & Grenier, D. (2016). Tea polyphenols inhibit the activation of NF‐κB and the secretion of cytokines and matrix metalloproteinases by macrophages stimulated with *Fusobacterium nucleatum* . Scientific Reports, 6(1), 1–11. 27694921 10.1038/srep34520 PMC5046134

[fsn33337-bib-0033] Li, R. , Huang, Y. G. , Fang, D. , & Le, W. D. (2004). (−)‐epigallocatechin Gallate inhibits lipopolysaccharide‐induced microglial activation and protects against inflammation‐mediated dopaminergic neuronal injury. Journal of Neuroscience Research, 78(5), 723–731. 10.1002/jnr.20315 15478178

[fsn33337-bib-0034] Li, S. , Lo, C. Y. , Pan, M. H. , Lai, C. S. , & Ho, C. T. (2013). Black tea: Chemical analysis and stability. Food & Function, 4, 10–18. 23037977 10.1039/c2fo30093a

[fsn33337-bib-0036] LIczbiński, P. , & Bukowska, B. (2022). Tea and coffee polyphenols and their biological properties based on the latest in vitro investigations. Industrial Crops and Products, 175, 114265. 34815622 10.1016/j.indcrop.2021.114265 PMC8601035

[fsn33337-bib-0037] Ma, Q. P. , Huang, C. , Cui, Q. Y. , Yang, D. J. , Sun, K. , Chen, X. , & Li, X. H. (2016). Meta‐analysis of the association between tea intake and the risk of cognitive disorders. PLoS One, 11(11), e165861. 10.1371/journal.pone.0165861 PMC5100989 27824892

[fsn33337-bib-0101] Maqbool, J. , Anwar, H. , Rasul, A. , Imran, A. , Saadullah, M. , Malik, S. A. , Shabbir, A. , Akram, R. , Sajid, F. , Zafar, S. , Saeed, S. , Akram, M. N. , Islam, F. , Hussain, G. , & Islam, S. (2023). Comparative evaluation of ethyl acetate and n‐Hexane extracts of *Cannabis sativa* L. leaves for muscle function restoration after peripheral nerve lesion. Food Science & Nutrition, 11(3), 1–9. 10.1002/fsn3.3255 PMC10261791 37324902

[fsn33337-bib-0038] Mathiyazahan, D. B. , Thenmozhi, A. J. , & Manivasagam, T. (2015). Protective effect of black tea extract against Aluminium chloride‐induced Alzheimer's disease in rats: A Behavioural, Biochemical and molecular approach. Journal of Functional Foods, 16, 423–435. 10.1016/j.jff.2015.05.001

[fsn33337-bib-0039] Maturana, M. G. V. , Pinheiro, A. S. , De Souza, T. L. F. , & Follmer, C. (2015). Unveiling the role of the pesticides Paraquat and rotenone on alpha‐synuclein fibrillation in vitro. Neurotoxicology, 46, 35–43. 10.1016/j.neuro.2014.11.006 25447323

[fsn33337-bib-0040] Mazumder, M. K. , & Choudhury, S. (2019). Tea polyphenols as multi‐target therapeutics for Alzheimer's disease: An in silico study. Medical Hypotheses, 125, 94–99. 10.1016/j.mehy.2019.02.035 30902161

[fsn33337-bib-0041] Modupe, O. D. , Samuel, O. O. , & Godwin, O. O. (2013). Trace metal concentrations in some tea leaves consumed in Ibadan, Nigeria. African Journal of Agricultural Research, 8(46), 5771–5775.

[fsn33337-bib-0042] Mushtaq, S. , Hina, S. , Maqbool, H. , Ahmed, A. , Nazim, M. , Hussain, E. , Mussab, R. M. , & Kumar, B. (2021). Frequency of peripheral nerve injury in trauma in emergency settings. Cureus, 13(3), e14195. 10.7759/cureus.14195 33948395 PMC8086758

[fsn33337-bib-0045] Rajha, H. N. , Paule, A. , Aragonès, G. , Barbosa, M. , Caddeo, C. , Debs, E. , … Edeas, M. (2022). Recent advances in research on polyphenols: Effects on microbiota, metabolism, and health. Molecular Nutrition & Food Research, 66(1), 2100670. 10.1002/mnfr.202100670 34806294

[fsn33337-bib-0046] Ramdani, D. , Chaudhry, A. S. , & Seal, C. J. (2018). Alkaloid and polyphenol analysis by HPLC in green and black tea powders and their potential use as additives in ruminant diets. In AIP conference proceedings 1927, 1, 030008. AIP Publishing LLC.

[fsn33337-bib-0047] Rana, A. , Samtiya, M. , Dhewa, T. , Mishra, V. , & Aluko, R. E. (2022). Health benefits of polyphenols: A concise review. Journal of Food Biochemistry, 46(10), e14264. 35694805 10.1111/jfbc.14264

[fsn33337-bib-0048] Rasul, A. , Al‐Shawi, A. A. , Malik, S. A. , Anwar, H. , Rasool, B. , Razzaq, A. , Aziz, N. , Kamran, S. A. S. , Sarfraz, I. , Shabbir, A. , Selamoglu, Z. , & Hussain, G. (2019). Neurada procumbens promotes functions regain in a mouse model of mechanically induced sciatic nerve injury. Pakistan Journal of Pharmaceutical Sciences, 32(4), 1761–1766. 31680070

[fsn33337-bib-0049] Razzaq, A. , Ahmad, S. , Farhan, M. , Ali, S. , Rasul, A. , Qasim, M. , Zafar, S. , Kamran, S. K. S. , Maqbool, J. , Imran, M. , Hussain, G. , & Hussain, M. (2020). *Moringa oleifera* Lam. Ameliorates the muscles function recovery following an induced insult to the sciatic nerve in a mouse model. Food Science & Nutrition, 8, 1–8. 10.1002/fsn3.1620 PMC7455924 32884682

[fsn33337-bib-0052] Sharma, A. , & Zhou, W. (2011). A stability study of green tea catechins during the biscuit making process. Food Chemistry, 126, 568–573.

[fsn33337-bib-0105] Shen, W. , Xiao, Y. , Ying, X. , Li, S. , Zhai, Y. , Shang, X. , Li, F. , Wang, X. , He, F. , & Lin, J. (2015). Tea consumption and cognitive impairment: a cross‐sectional study among Chinese elderly. PLoS One, 10(9), e0137781. 26359663 10.1371/journal.pone.0137781 PMC4567322

[fsn33337-bib-0053] Stangl, V. , Dreger, H. , Stangl, K. , & Lorenz, M. (2007). Molecular targets of tea polyphenols in the cardiovascular system. Cardiovascular Research, 73, 348–358. 10.1016/j.cardiores.2006.08.022 17020753

[fsn33337-bib-0054] Sun, S. , Pan, S. , Ling, C. , Miao, A. , Pang, S. , Lai, Z. , Chen, D. , & Zhao, C. (2012). Free radical scavenging abilities in vitro and antioxidant activities in vivo of black tea and its main polyphenols. Journal of Medicinal Plants Research, 6(1), 114–121.

[fsn33337-bib-0056] Tysnes, O. B. , & Storstein, A. (2017). Epidemiology of Parkinson's disease. Journal of Neural Transmission, 124(8), 901–905. 10.1007/s00702-017-1686-y 28150045

[fsn33337-bib-0057] Wang, H. , Ding, X.‐G. , Li, S.‐W. , Zheng, H. , Zheng, X.‐M. , Navin, S. , Li, L. U. , & Wang, X.‐H. (2015). Role of oxidative stress in surgical cavernous nerve injury in a rat model. Journal of Neuroscience Research, 93(6), 922–929. 10.1002/jnr.23545 25597854

[fsn33337-bib-0106] Xie, B. , Shi, H. , Chen, Q. , Ho, C.T. (1993). Antioxidant properties of fractions and polyphenol constituents from green, oolong and black teas. Proceedings of the National Science Council, Republic of China, 17(2): 77‐84. 7809277

[fsn33337-bib-0058] Yang, Z. , Jie, G. , Dong, F. , Xu, Y. , Watanabe, N. , & Tu, Y. (2008). Radical‐scavenging abilities and antioxidant properties of theaflavins and their gallate esters in H_2_O_2_‐mediated oxidative damage system in the HPF‐1 cells. Toxicology In Vitro, 22(5), 1250–1256. 18502093 10.1016/j.tiv.2008.04.007

[fsn33337-bib-0059] Zhou, Z. D. , Xie, S. P. , Saw, W. T. , Ho, P. G. H. , Wang, H. , Lei, Z. , Yi, Z. , & Tan, E. K. (2019). The therapeutic implications of tea polyphenols against dopamine (DA) neuron degeneration in Parkinson's disease (PD). Cells, 8(8), 911. 10.3390/cells8080911 31426448 PMC6721683

[fsn33337-bib-0060] Zhu, W. L. , Shi, H. S. , Wei, Y. M. , Wang, S. J. , Sun, C. Y. , Ding, Z. B. , & Lu, L. (2012). Green tea polyphenols produce antidepressant‐like effects in adult mice. Pharmacological Research, 65(1), 74–80. 10.1016/j.phrs.2011.09.007 21964320

